# Multi-channel EEG recording during motor imagery of different joints from the same limb

**DOI:** 10.1038/s41597-020-0535-2

**Published:** 2020-06-19

**Authors:** Xuelin Ma, Shuang Qiu, Huiguang He

**Affiliations:** 1 The Research Center for Brain-Inspired Intelligence & National Laboratory of Pattern Recognition, Institute of Automation, Chinese Academy of Sciences (CASIA), Beijing, 100190 China; 2 The School of Artificial Intelligence, University of Chinese Academy of Sciences, Beijing, 100049 China; 3 The Center for Excellence in Brain Science and Intelligence Technology, Chinese Academy of Sciences, Beijing, 100190 China

**Keywords:** Brain imaging, Biomedical engineering

## Abstract

Motor imagery (MI) is one of the important brain-computer interface (BCI) paradigms, which can be used to control peripherals without external stimulus. Imagining the movements of different joints of the same limb allows intuitive control of the outer devices. In this report, we describe an open access multi-subject dataset for MI of different joints from the same limb. This experiment collected data from twenty-five healthy subjects on three tasks: 1) imagining the movement of right hand, 2) imagining the movement of right elbow, and 3) keeping resting with eyes open, which results in a total of 22,500 trials. The dataset provided includes data of three stages: 1) raw recorded data, 2) pre-processed data after operations such as artifact removal, and 3) trial data that can be directly used for feature extraction and classification. Different researchers can reuse the dataset according to their needs. We expect that this dataset will facilitate the analysis of brain activation patterns of the same limb and the study of decoding techniques for MI.

## Abstract (summary)

**Table:** 

Measurement(s)	brain measurement • functional brain measurement
Technology Type(s)	electroencephalography (EEG) • silver/silver chloride reference electrode
Factor Type(s)	type of motion imagery task
Sample Characteristic - Organism	Homo sapiens

Machine-accessible metadata file describing the reported data: 10.6084/m9.figshare.12278444

## Background & Summary

Brain-computer interface (BCI) system establishes a direct communication and control path between the brain and the external devices that does not depend on the peripheral nerves and muscles^1^. Electroencephalography (EEG) has been widely used for BCI because of its high temporal resolution, cost-effectiveness, portability and noninvasive nature^2^. Compared with other BCI paradigms, such as steady state visual evoked potentials (SSVEPs)^3^, P300 potentials^4^, etc., a motor imagery (MI)-based BCI can be independent of external stimulus and reflect the subject’s voluntary movement awareness^5^.

Most existing MI studies focus on different limbs (e.g. left hand, right hand)^6–8^ or their combinations (e.g. both left hand and feet)^9,10^. The BCI systems based on the MI of different limbs have been successfully applied to control wheelchair^11^ and mechanical exoskeleton^12,13^, as well as for post stroke rehabilitation^14,15^. However, imagining the movement of different upper limbs to control a peripheral device to perform different actions will lead to an inconsistency between the intention of movement and the action of the end effector, which is called cognitive disconnection^16^. For example, it was unnatural to open and close the hand orthosis by imagination of right hand and both feet movement respectively^6^. Therefore, the decoding of fine imaging movement from the same limb has aroused more and more concern.

In the early stage of the study, most researchers focus on the fine movement of one single joint. For examples, Salehi *et al*.^17^ and Alazrai *et al*.^18^ did some research on motor imagery recognition of figure gestures. Vuckovic and Sepulveda utilized Gabor transform features for decoding four wrist movements (flexion, extension, pronation, and supination)^19^. Afterwards, Edelman *et al*. improved the decoding accuracy of these wrist imagining movements from 69.1% to 81.4% by source space analysis^16^. Discriminating the MI of different joints is also essential for general intuitive control of the limb. However, there are few studies on this aspect, and the decoding accuracy cannot meet the requirements of practical application. Suwannarat *et al*. developed a motor imagery training system with three different joint movements of same limb, namely, the hand opening/closing, wrist flexion/extension and forearm pronation/supination^20^. In 2015, Yong *et al*. first investigated a 3-class BCI system for rest, imaginary grasp and elbow movement with an averaged classification accuracy of 60.7%^21^. More recently, Tavakolan *et al*. reported that the accuracy can be improved to 74.2% by extracting time-domain features^22^.

In this paper, we collected data from twenty-five subjects in the new dataset MI-2, which contains a total of 22,500 (=25 · 900) hand movement MI, elbow movement MI, and resting state trials. The subjects performed specific MI tasks according to the prompts on the screen and their EEG data were recorded at the same time. During the experiment, EMG signals of the limbs were monitored to make sure that the collected EEG data were indeed the result of motor imagery, not motor execution. The public dataset consists of three stages of data, namely the raw data, the pre-processed data, and the trial data that can be directly used for classification, so that different researchers can reuse the dataset according to their needs.

Based on this dataset, we have preliminarily compared the differences in EEG activation patterns between hand and elbow MI, and conducted a three-classification decoding task using the existing baseline and the state-of-the-art methods, proving that the collected data of the three categories are separable^23^. Also, we extracted the correlation coefficient matrices between channels as features representing functional connections, and developed an ensemble Channel-Correlation Network to improve the decoding performance. In this dataset, our proposed deep learning based method obtained a decoding accuracy of 87.03% in the 3-class scenario^23^.

This paradigm of different joints from the same limb can provide intuitive control of outer equipments (e.g. exoskeleton), without the need to increase the cognitive load of the users to establish any artificial associations between imaging movement and neuroprosthetic movement. We expected that this dataset could be used to analysis the brain activation patterns and to design the decoding methods of MI of different joints from the same limb.

## Methods

### Subjects

All experiments were approved by the ethical committee of Institute of automation, Chinese Academy of Sciences. In this experiment, we collected data from 25 right-handed healthy subjects (19 males, 6 females, aged 19–27) without MI-based BCI experience. All subjects signed the informed consent before the experiment. The names of all participants have been hereby anonymized. The participants are identified only by their aliases “sub-001” through “sub-025”.

### Experimental paradigm

**Figure 1:**
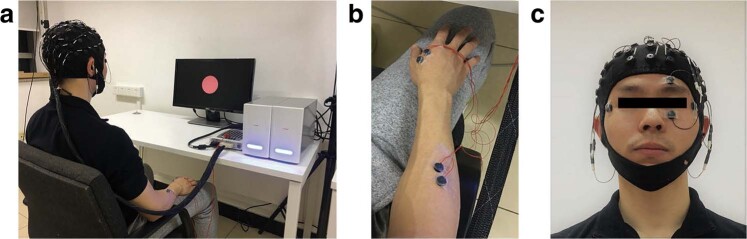
Data acquisition scenario. Informed consent was obtained from the individual in the figure for the publication of the images. (**a**) The Neuroscan SynAmps2 amplifier (Neuroscan, Inc.) with a 64-channel electrode cap according to the standard 10/20 System. (**b**) The EMG of the forearm and upper arm of the right arm were monitored. (**c**) Horizontal and vertical EOGs were collected for later artifact removal.

**Figure 2:**
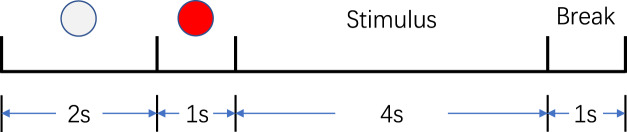
Timing of one trial.

The subjects sat in a comfortable chair with their hands naturally on their thighs, keeping their eyes one-meter away from the screen (see Fig. 1(a)). As shown in Fig. 2, each trial (8s) started with a white circle at the center of the monitor for 2s, followed by a red circle as a cue for 1s to remind the subjects of paying attention to the upcoming target. The target prompt (“Hand” or “Elbow”) appeared on the screen for 4s. During this period, the subjects were asked to imagine the prompted movement kinesthetically in mind rather than a visual type of imagery. The subjects were instructed to avoid any motion during imagination. The EMG of the right hand and the right forearm of the subjects were monitored (see Fig. 1(b)) to make sure they did not move involuntarily. After the imagination, “Break” appeared for 1s as the end of the entire 8s trial. During the break, the subjects were asked to relax and minimize their eye and muscle movements.

**Figure 3:**
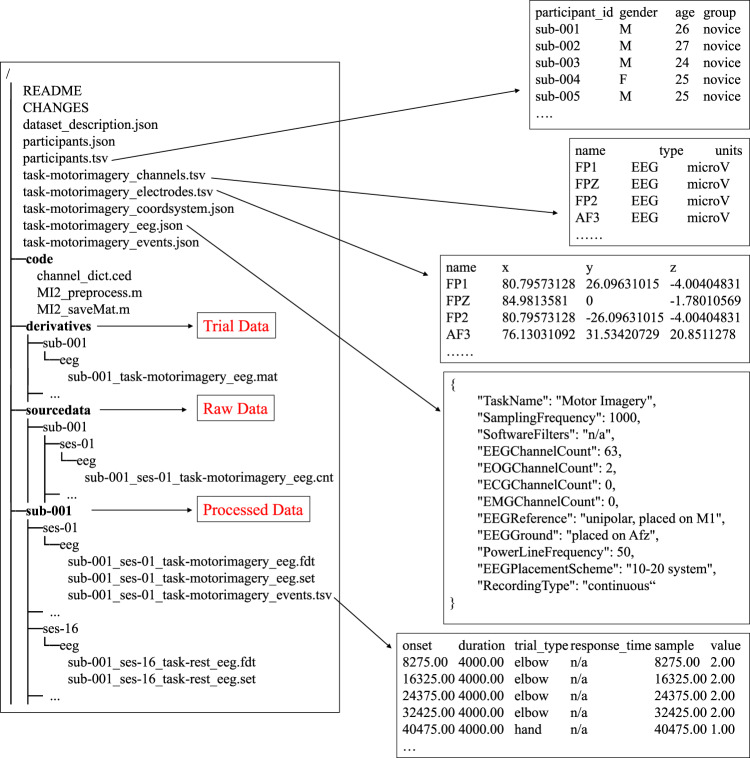
Directory tree for the repository with previews of EEG files. The left side of the figure is the directory tree for our repository and the arrows indicate the contents of the folder or preview contents of the meta-data files.

**Figure 4:**
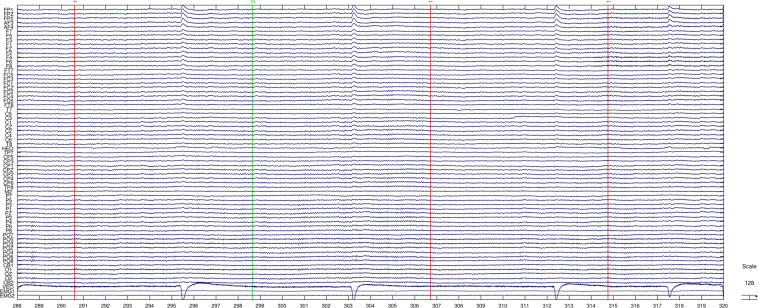
Channel activities snapshot of the collected raw EEG signals (.cnt file). The bottom two channels are EMG channels.

**Figure 5:**
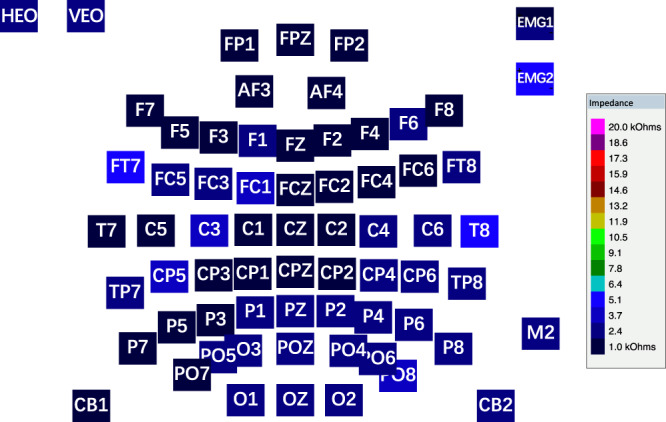
The impedances of all the 65 electrodes were kept close to or below 5 kilo-ohms.

**Figure 6:**
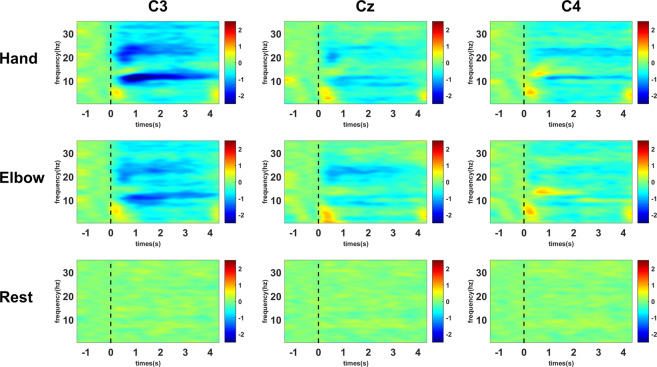
Averaged time-frequency maps of 25 subjects. Blue indicates ERD.

**Figure 7:**
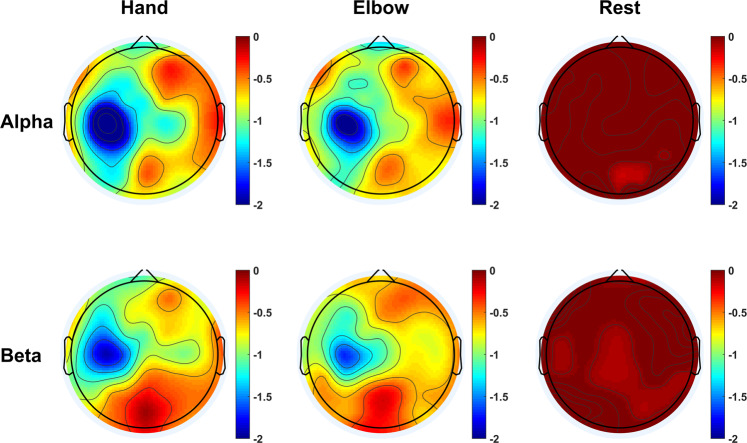
Averaged topographical distribution of power of 25 subjects in the upper alpha band (10–12 Hz) and upper beta band (23–25 Hz). Blue indicates ERD.

**Figure 8:**
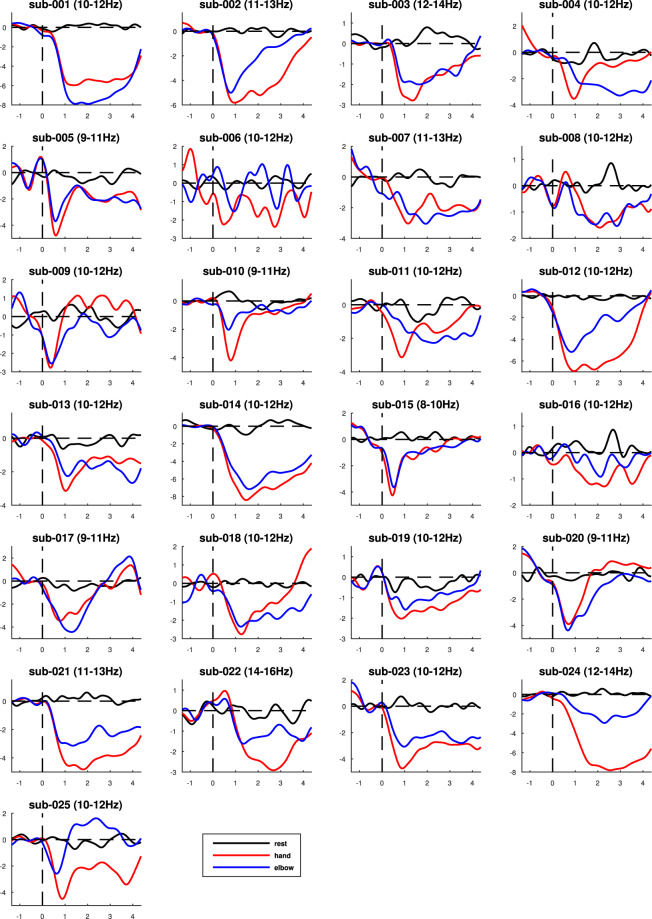
The comparison of power (in dB) changes with time (in s) during hand, elbow motor imagery, and resting state for electrode C3. The specific frequency band of each subject is indicated in the title.

The experiments contained 7 sessions, involving five sessions consisting of 40 trials each for two kinds of motor imagination tasks (20 trials for each movement imagination in one session) and two sessions consisting of 50 trials each for resting state. In order to avoid interference between the imaginary tasks before and after, the order of the MI task indications in a session was disrupted. There were 5 to 10 minutes of rest between sessions. Thus, there are totally 300 trials (100 trials for each type of mental state) per subject in the dataset.

### Data collection and preprocessing

As shown in Fig. 1(a), EEG data were acquired using a 64-channel gel electrode cap (according to the standard 10/20 System) with a Neuroscan SynAmps2 amplifier (Neuroscan, Inc.). The sampling frequency was 1000 Hz. The left mastoid reference was used for EEG measurement. Electrode impedances were kept below 10 *k*Ω during the experiment. The band-pass filtering range of the system was 0.5–100 Hz. Besides, an additional 50 Hz notch filter was used for data acquisition.

The pre-processing of the collected data was done using the EEGLAB toolkit (v14.1.1_b)^24^ of MATLAB (R2015a) software. We used Common Average Reference (CAR) to spatially filter the data, and performed time-domain filtering on the data from 0 to 40 Hz. The formula of CAR is shown in Eq. (1), where *N* represents the number of EEG channles. The data were downsampled to 200 Hz to reduce computational cost. A plugin in EEGLAB called Automatic Artifact Removal toolbox (AAR)^25^ was used to automatically remove the ocular and muscular artifacts in EEG.

1 $${\widetilde{s}}_{i}={s}_{i}-\frac{1}{N}\mathop{\sum }\limits_{i=1}^{N}{s}_{i}$$

## Data Records

The data are freely downloaded from the open access repository^26^. The source files and meta-data files in this dataset were organized according to EEG-BIDS^27^, which was an extension to the brain imaging data structure for EEG. The directory tree for our repository and some previews for meta-data are shown in Fig. 3.

On the whole, the repository’s data consists of three parts: (1) Preprocessed Data stored in the home folder – *set/fdt* files with meta-data; (2) Raw Recorded Data stored in the *sourcedata* folder – *cnt* files; (3) Trial Data stored in the *derivatives* folder – *mat* files. Within these directories, the subdirectory corresponding to each subject is named “sub-xxx”, where xxx represents the serial number of the subject.

### Raw Data

The acquired raw data for a single task session were saved as .cnt files and organized according to the following naming rules:

$$sub-xxx\_ses-yy\_task-TASKNAME\_eeg.cnt,$$

where *xx* stands for the subject number (001, 002, …, 025), *yy* is the session number (01, 02, …, 19), *TASKNAME* represents the task type (“motorimagery” or “rest”).

After loading the.cnt file through EEGLAB toolbox, you will get a structure variable named “EEG” whose key field information can be seen in Table 1.

**Table 1:** Contents of EEG structure in.cnt and set/.fdt files.

Name	Description
EEG.nbchan	The number of channels
EEG.srate	The sampling rate of data
EEG.pnts	The total number of sampling points
EEG.data	(EEG.nbchan · EEG.pnts) matrix containing the EEG data
EEG.chanlocs	Information about the names of the channels and their corresponding locations. For example, EEG.chanlocs(33) and EEG.chanlocs(65) represent Horizontal EOG (HEO) and Vertical EOG (VEO) respectively. Note that the channel locations are only available in .set/.fdt files.
EEG.urevent	Records of when the event occurred.
EEG.urevent.type	“1” represents hand movement and “2” represents elbow movement
EEG.reject	Statistics used for data rejection

**Table 2:** Classification accuracy obtained with FBCSP + SVM method.

Subject	Acc. (%)	Subject	Acc. (%)	Subject	Acc. (%)
sub-001	69.00	sub-010	70.78	sub-019	62.44
sub-002	68.11	sub-011	68.89	sub-020	67.78
sub-003	71.67	sub-012	71.67	sub-021	69.67
sub-004	69.00	sub-013	67.67	sub-022	69.89
sub-005	66.33	sub-014	67.44	sub-023	66.78
sub-006	73.44	sub-015	71.33	sub-024	70.22
sub-007	63.78	sub-016	69.11	sub-025	68.00
sub-008	67.33	sub-017	67.78	mean	68.68
sub-009	71.89	sub-018	67.11	std	2.44

### Processed Data

Raw data (.cnt files) from Neuroscan was imported to MATLAB using the EEGLAB toolbox. After the preprocessing operations such as re-referencing and artifact removal were performed, the data of each session were saved as two associated files: FILENAME.set and FILENAME.fdt (.fdt for float data). Note that at this stage the two EMG channels that were not relevant to the EEG data analysis were removed. The name of them are consistent with the raw recorded data except for the suffix:

$$sub-xxx\_ses-yy\_task-TASKNAME\_eeg.set(.fdt),$$

The .set and .fdt files store meta information (e.g. number of channels, sampling rate) and raw data respectively.

After loading the .set/.fdt file through EEGLAB toolbox, you will get a structure variable named “EEG” whose key field information can be seen in Table 1.

### Trial Data

To facilitate the decoding of movement intentions, the trial data of each subject were integrated into a single .mat file, which are ready for feature extraction and classification. This folder contains 25 .mat files named in the format *sub* – *xxx*_*task* – *motorimagery*_*eeg*.*mat*, where *xxx* stands for the subject number (001, 002, …, 025). Each.mat file stores three variables:

- task_data: It contains all the task data of a subject with a size of (session number, trial number, channel number, sample points), e.g. (15, 40, 62, 800).
- task_label: It contains all the task label (“1” and “2” for MI of hand and elbow, respectively) of a subject with a size of (session number, trial number), e.g. (15, 40).
- rest_data: It contains a total of 300 trials from 4 rest sessions (75*4) with a size of (trial number, channel number, sample points), e.g. (300, 62, 800).

## Technical Validation

### EMG Validation

During imagination, the subjects were instructed to avoid any motion. The electromyography (EMG) signal snapshot is shown in Fig. 4 (the bottom two channels). When we observed actual hand movements or fluctuations in EMG signals, we would remind the subject and restart the session. In this way, we can ensure that the collected EEG is indeed the result of motor imagery, not motor execution. Due to equipment problems, the EMG signals were only available in the last 15 subjects (from sub-011 to sub-025).

### EEG Validation

As shown in Fig. 5, during the experiment, the impedances of electrodes were kept close to or below 5 kilo-ohms to ensure the quality of the connections between the electrodes and the scalp.

Event related desynchronization (ERD, reduction in power) in the alpha and beta band during the period of motor imagery has been extensively documented in the literature^28,29^. This characteristic behaviors is observed in our data. Figure 6 shows the average time-frequency maps of all subjects at electrode positions C3, C4 and Cz during the MI of hand, elbow, and resting state. The maps show clear reduction of power in both alpha (8–13 Hz) and beta (20–25 Hz) bands from 0 to 4s, except for resting state. In order to study the activation of the motor area, we calculated the average energy distribution in the alpha and beta bands, and plotted them into a topology map based on the channel positions, which are depicted in Fig. 7. The maps of the hand and elbow MI show a clear ERD pattern in the contralateral motor areas, while that of the resting state diagram does not.

In addition, we determined a 2Hz-wide frequency band according to the frequency of the lowest energy position in each subject’s ERD graph, and plotted the curve of energy with time. As shown in Fig. 8, the power decrease after the onset (0-s on the x-axis) of the motor imagery.

These results indicate that in our experiments, subjects’ imagination of the hands and elbows activated their corresponding areas of motion on the opposite side of the brain, which is consistent with previous studies^28^.

In addition to the above qualitative analysis, there is also quantitative analysis. We extracted the features of FBCSP^30^ and used the SVM as our classifier to classify the data of each subject. Table 2 shows the classification accuracies of cross-trial under 5-fold cross-validation. The classification results of each subject were above the chance level (33.33%) in the 3-class scenario.

## Usage Notes

The raw recorded EEG dataset, the pre-processed dataset, and the classification-ready trial-wise dataset are available from Harvard Dataverse^26^. The data can be analyzed in MATLAB using the EEGLAB toolkit. Detailed tutorials of EEGLAB can be found on their website (https://sccn.ucsd.edu/eeglab/index.php). Although the results may not vary much between versions, we recommend the researchers use EEGLAB with version v14.1.1_b.

We provide some instructions on how to use EEGLAB to load our data.

1. Load the **.cnt** file. First select “File” from the menu, then select the sub-menu “Import data”, “Using EEGLAB functions and plugins”, “From Neuroscan .CNT file”, select the target CNT file (e.g. sub-001_ses-01_task-motorimagery_eeg.cnt) from the file navigation window that pops up, and click “OK”.
2. Load the **.set** file. First select “File” from the menu, then select the sub-menu “Load existing dataset”, select the target SET file (e.g. sub-001_ses-01_task-motorimagery_eeg.set) from the file navigation window that pops up, and click “OK”.
3. Check the workspace in MATLAB. The “EEG” variable contains the information as shown in Table 1.

## Data Availability

The custom MATLAB script *MI2_preprocess.m* was used to pre-process the raw recorded data. The *MI2_saveMat.m* file was used to extracted the 4s-trial data from continuous data and save them to the.mat files. The *channel_dict.ced* file contains the location information of channels for our EEG record data and can be easily imported into EEGLAB. These scripts and files are stored in the code folder and shared with the dataset.
